# The Influence of Uric Acid Concentration on the Daily Functioning of Patients at an Advanced Age, Based on the Results of Selected Point Scales Routinely Used for the Comprehensive Geriatric Assessment in Poland

**DOI:** 10.3390/jcm14165793

**Published:** 2025-08-15

**Authors:** Jakub Husejko, Mariusz Kozakiewicz, Marcin Gackowski, Katarzyna Mądra-Gackowska, Jakub Wojtasik, Iga Hołyńska-Iwan, Mateusz Porada, Magdalena Kiełkucka, Karol Harmoza, Anna Pokrzywa, Maja Kubiaczyk, Albert Jaśniak, Kornelia Kędziora-Kornatowska

**Affiliations:** 1 Department of Geriatrics, Faculty of Health Science, L. Rydygier Collegium Medicum in Bydgoszcz, Nicolaus Copernicus University in Torun, Skłodowska-Curie 9 Street, 85-094 Bydgoszcz, Poland; markoz@cm.umk.pl (M.K.); katarzyna.madra@cm.umk.pl (K.M.-G.); poradamatt@gmail.com (M.P.); magdakielk@gmail.com (M.K.); harmoz.k@gmail.com (K.H.); pokrz.anna@gmail.com (A.P.); majakkubiaczyk@gmail.com (M.K.); kornelia.kornatowska@cm.umk.pl (K.K.-K.); 2 Department of Toxicology and Bromatology, Faculty of Pharmacy, L. Rydygier Collegium Medicum in Bydgoszcz, Nicolaus Copernicus University in Torun, A. Jurasza 2 Street, 85-089 Bydgoszcz, Poland; marcin.gackowski@cm.umk.pl; 3 Centre for Statistical Analysis, Nicolaus Copernicus University in Toruń, Chopina 12/18 Street, 87-100 Toruń, Poland; jwojtasik@umk.pl; 4 Department of Pathobiochemistry and Clinical Chemistry, Faculty of Pharmacy, Ludwik Rydygier Collegium Medicum in Bydgoszcz, Nicolaus Copernicus University in Torun, 85-094 Bydgoszcz, Poland; 5 Department of Cardiology and Clinical Pharmacology, Faculty of Health Sciences, Collegium Medicum in Bydgoszcz, Nicolaus Copernicus University, 87-100 Torun, Poland; albertjasniak@gmail.com

**Keywords:** uric acid, older adults, cognitive function, nutritional status, geriatric scales, comprehensive geriatric assessment, ADL, MMSE, ACE-III, GDS

## Abstract

**Background/Objectives**: The concentration of uric acid in the body of older adults may have various effects. Due to the multidirectional influence on metabolism, its significance in the daily functioning of older persons remains unclear. The present investigation explored whether serum uric acid levels are associated with scores on standard geriatric assessment scales in hospitalized older adults. **Methods**: In total, 77 patients admitted to the hospital for the Comprehensive Geriatric Assessment were recruited and classified into three groups: successfully treated for hyperuricemia, untreated or unsuccessfully treated with elevated uric acid levels, and untreated controls having normal uric acid levels. The analysis considered the relationship between the concentration of uric acid in patients from different study groups and the assigned classes defined by the ranges of the questionnaires used for the study. **Results**: Significant differences were shown in the distribution of classes defined by Addenbrooke’s Cognitive Examination III (ACE-III) and the MNA questionnaires concerning the study groups. Moreover, significant differences were confirmed when using compartmentalization based only on the screening test results for the ACE-III, the Mini Nutritional Assessment (MNA), and the Mini-Mental State Examination (MMSE). For ACE-III, a lower percentage of people with probable dementia was observed in the control group (34.5%) than in the group with elevated uric acid values (78.3%). **Conclusions**: Although the mechanisms related to uric acid’s influence on older people’s functioning require further research, the available evidence indicates a negative impact of elevated uric acid levels on cognitive functions and the nutritional status of older individuals.

## 1. Introduction

Uric acid is the end product of purine metabolism, a chemical compound naturally occurring in living organisms. Purines are components of cells and play a key role in biochemical processes such as DNA and RNA synthesis. In a healthy organism, the uric acid level in the blood is 3.5–7.2 mg/dL. If the concentration exceeds 7.2 mg/dL, we talk about hyperuricemia, i.e., increased levels of uric acid in the blood [1]. The concentration of uric acid in the body of older adults can have various effects. Interestingly, high-normal uric acid levels in the blood show antioxidant properties. However, hyperuricemia has adverse effects, leading to a decrease in the level of independence, as well as an increased risk of depression in old age [2]. Over the past two decades, the narrative around uric acid has moved beyond its traditional identification as a mere precursor of gout toward appreciation of its pleiotropic, context-dependent actions in aging, ranging from antioxidant neuroprotection to pro-oxidant and pro-inflammatory effects [3,4]. This evolution parallels the historical shift in lipid research from total-cholesterol measurements to the nuanced assessment of lipoprotein subclasses. It also highlights the need for similarly sophisticated approaches when evaluating UA in geriatric outcomes.

Some studies suggest that elevated uric acid levels may protect neurons from oxidative stress, potentially reducing the risk of dementia and Parkinson’s disease. Scientists have shown that elevated serum uric acid levels contribute to improved cognition and reduced risk of dementia [2]. It is observed that older people with slightly higher levels of this compound often demonstrate better cognitive functions than their peers with lower levels of uric acid. High-normal uric acid levels contribute to better physical fitness, muscle strength, and overall functioning in older people [5,6]. It has been studied that hemodialysis patients with hyperuricemia were better nourished, while patients with normal uric acid levels were more susceptible to malnutrition and cachexia [7].

It is worth noticing that hyperuricemia is strongly associated with many diseases that mainly affect seniors [8]. It increases the risk of hypertension and insulin resistance by damaging the beta cells of the pancreas that produce insulin [9], type 2 diabetes, hypothyroidism [10], neurodegenerative diseases such as Alzheimer’s disease [11], and cardiovascular diseases [12]. In addition, excess uric acid leads to gout, a disease manifested by painful inflammation of the joints, which also limits seniors’ mobility and independence. A negative correlation has been shown between hyperuricemia and the occurrence of symptoms of depression in people over 60 years of age, especially in the group of postmenopausal women [13].

Maintaining an appropriate uric acid balance may be critical for late-life health. It could guide future diagnostic and therapeutic strategies by clarifying its dual harmful and protective roles. The present contribution explored whether serum uric acid concentration is linked to outcomes on commonly used geriatric scales in a cohort of hospitalized older patients. The analysis considered the relationship between the concentration of uric acid and the final results of selected scales commonly used in Polish geriatric wards.

## 2. Materials and Methods

The analysis included 77 patients hospitalized in the Department of Geriatrics and Internal Medicine of the University Hospital No. 1, named after Dr. A. Jurasz in Bydgoszcz (Poland) between 1 July 2022 and 31 December 2023. The study participants were then divided into three groups:

1. Patients treated for hyperuricemia who had normal uric acid levels at recruitment;
2. Patients with elevated uric acid levels during recruitment—untreated or unsuccessfully treated;
3. Control group—patients not treated for hyperuricemia and with normal uric acid levels.

Patients meeting the following criteria were excluded from the study: age < 60 years; incapacitated persons, conscript soldiers, deprived of liberty, in a service-related or other dependency with the researcher; diagnosed with profound dementia; participant withdrawal from the study. Uric acid levels were measured at the beginning of each patient’s hospitalization using the enzymatic method, with reference values from 2.5 to 6.2 mg/dL. Geriatric scales were performed in the final days of hospitalization after stabilizing the general condition. The study was approved on 14 December 2021, by the Bioethics Committee of the Collegium Medicum of the Nicolaus Copernicus University in Toruń, application number KB 675/2021.

The following point scales were used to assess physical health, functional and nutritional status, mental state, and socio-environmental situation during the hospital stay:

1. The ADL (Activities of Daily Living) is a questionnaire assessing the ability of the examined person to perform activities that allow independent coping with basic needs, including bathing, dressing, using the toilet, moving, controlled excretion of urine and stool, and eating. For independent performance of the above activities, 1 point is awarded; if they cannot do it, they receive 0 points. A maximum of 6 points can be awarded, which means full functionality and a minimum of 0. Obtaining less than 3 points means severe disability [14].
2. The MMSE (Mini-Mental State Examination) scale is a tool used to assess the patient’s cognitive functions. It contains 30 questions covering various cognitive function areas, including orientation, memory, attention, calculation, memorization, language, and following commands. The MMSE scale results can range from 0 to 30 points. The higher the number of points, the better the patient’s cognitive function. Values below 24 points may suggest the presence of cognitive disorders, such as dementia, but the final diagnosis requires further tests [15].
3. The ACE-III (Addenbrooke’s Cognitive Examination III) scale is a diagnostic tool also used to assess cognitive functions. However, it is more detailed than the MMSE. It consists of 5 main areas of assessment, which cover different aspects of cognitive functions: orientation, memory, attention, calculation, language, and visual–spatial skills. The total number of points obtained in the ACE-III scale is 100 [16]. Polish normative data for adults aged ≥65 years indicate that scores below 88/100 fall below the 5th percentile, suggesting cognitive impairment [17]. Accordingly, we classified participants with ≤87 points as cognitively impaired.
4. The Mini Nutritional Assessment (MNA) is a diagnostic tool used to assess the nutritional status of older people. It consists of 18 questions divided into two main parts: nutritional assessment (6 questions) and assessment of anthropometric measurements (12 questions). Based on the answers, the MNA scale score is calculated, and the patient receives one of three possible results: no malnutrition, risk of malnutrition, or malnutrition [18].
5. The GDS (Geriatric Depression Scale) is a scale that allows screening for the intensity of depression symptoms in older adults. The questionnaire consists of 30 questions that can be answered with short “yes” or “no” answers. The questions concern the feelings of the examined person during the last week. The answers are additionally marked with asterisks. For each marked answer with an asterisk, the patient receives 1 point. The more points obtained, the greater the risk of severe depression [19].

Depending on data distribution, one-way ANOVA or a Kruskal–Wallis test was applied after checking normality (Shapiro–Wilk test) and homogeneity of variance (Levene test). In the case of statistically significant differences in subgroups, Tukey’s post hoc tests (when parametric ANOVA was performed) or Mann–Whitney U tests (when the Kruskal–Wallis test was performed) were performed with Bonferroni’s *p*-value correction for multiple testing. Neyman’s chi-square tests of independence were used to compare scales as qualitative variables, and, in the case of failure to meet the appropriate assumptions regarding the expected frequencies in subgroups, Fisher–Freeman–Halton tests were used to compare scales. The Python programming language (v. 3.10.6) [20] was used to perform the analyses with the following libraries: matplotlib (v. 3.10.0) [21], numpy (v. 2.2.1) [22], pandas (v. 2.2.3) [23], pingouin (v. 0.5.5) [24], tableone (v. 0.9.1) [25], seaborn (v. 0.13.2) [26]. All analyses were performed, assuming a significance level of 0.05. The power of the tests was estimated using the Monte Carlo method with 10,000 repetitions.

## 3. Results

### 3.1. Descriptive Statistics by Groups of Subjects

The present cross-sectional observational study included 77 older inpatients, of whom 45 were female and 32 were male. In total, 25 patients were successfully treated for hyperuricemia, 23 had elevated uric acid levels, and 29 were assigned to the control group. Descriptive statistics for quantitative variables are presented in Table 1.

Screening tests from the Comprehensive Geriatric Assessment captured four key domains: daily functioning, cognition, nutritional status, and depressive symptoms. To show a relationship between established groups and patients’ condition, the examined patients were additionally assigned to classes corresponding to the classification based on a screening tool’s score range (Table 2). The results that were obtained underline the problematic situation of Polish senile patients. First of all, almost half of the studied population could not independently cope with basic needs; similarly, more than half of the examined inpatients suffered from cognitive impairments or dementia. According to the MNA questionnaire, among 77 recruited senile inpatients, 10 were malnourished, 37 were at risk of malnutrition, and 30 had a normal nutritional status. It corresponds to 13.00%, 48.10%, and 39.00% of the studied population. That means that 61% of older hospitalized patients were at risk of nutrition-related complications. Finally, according to the GDS, depression is prevalent in 51.90% of the examined patients.9n

Statistical tests were performed to verify the significance of differences between the distributions of classes in each of the study groups. For this purpose, chi-square tests of independence were used (*p*-value written in the standard font) or, in the case of too low frequencies in subgroups, Fisher–Freeman–Halton exact tests (FFH, *p*-value written in italics). Significant differences were shown in the distribution of classes defined by the ACE-III and the MNA questionnaires concerning the study groups (*p*-values of 0.007 and 0.044, respectively). Chi-square tests of independence or FFH were performed again with the Benjamini–Hochberg correction for multiple testing to indicate which group differences in the distribution of classes were observed. The results are presented in Table 3. For the ACE-III scale, statistically significant differences were demonstrated in the distribution of classes between the control group and patients with elevated uric acid levels. In the control group, a lower percentage of people with probable dementia was observed (34.5%) than in the group with elevated uric acid values (78.3%). Based on the above, there is a correlation between the result obtained on the ACE-III and uric acid levels described by affiliation in one of the three groups designed in the study. For the MNA questionnaire, statistically significant differences were demonstrated in the distribution of classes between the control group and patients with elevated uric acid levels, and between patients with elevated uric acid levels and patients properly treated for hyperuricemia. The risk of nutrition-related complications was significantly higher for individuals in the elevated UA levels group, because only 21.7% of individuals had an adequate nutritional status, contrary to over half of the controls. Similarly, the risk of malnutrition was significantly higher among patients treated adequately for hyperuricemia in comparison to patients untreated or inappropriately treated.

### 3.2. Uric Acid Values by Geriatric Scales

Differences between the groups of patients classified according to the results of screening tests performed were observed for MMSE, ACE-III, and MNA questionnaires. The results of the ANOVA and Kruskal–Wallis tests are shown in Table 4. The Kruskal–Wallis test was considered when parametric methods demonstrated no statistically significant differences.

#### 3.2.1. Uric Acid and the MMSE

**Figure 1:**
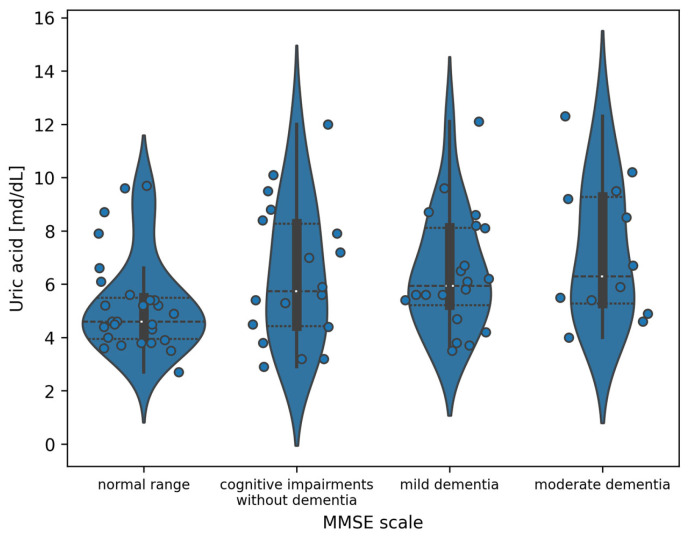
Violin plot of the distribution of uric acid measurement values in the division of patients according to the MMSE (white dots indicate the median value).

For the MMSE, the most significant differences are observed between the normal range and moderate dementia subgroups. The entire distribution of uric acid values divided into classes defined by the MMSE is presented in Figure 1. However, using Mann–Whitney U tests with Benjamini–Hochberg correction does not allow us to state that these differences are statistically significant (*p* = 0.660). The full results of post hoc tests are included in Table 5.

#### 3.2.2. Uric Acid and the ACE-III

**Figure 2:**
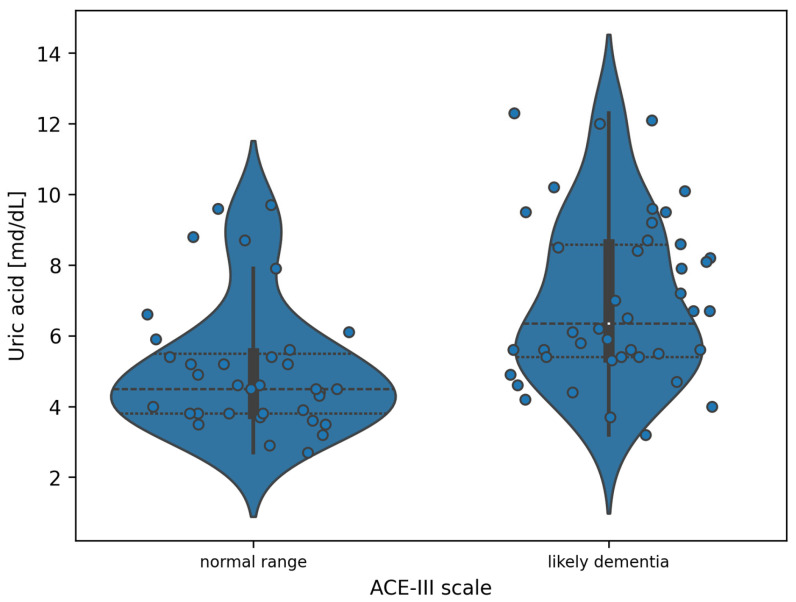
Violin plot of the distribution of uric acid measurement values in the division of patients according to the ACE-III (white dots indicate the median value).

Due to the two-class nature of the ACE-III, a statistically significant ANOVA test result is equivalent to the existence of statistically significant differences in uric acid values between the subgroups. The exact differences are presented in Figure 2.

#### 3.2.3. Uric Acid and the MNA

**Figure 3:**
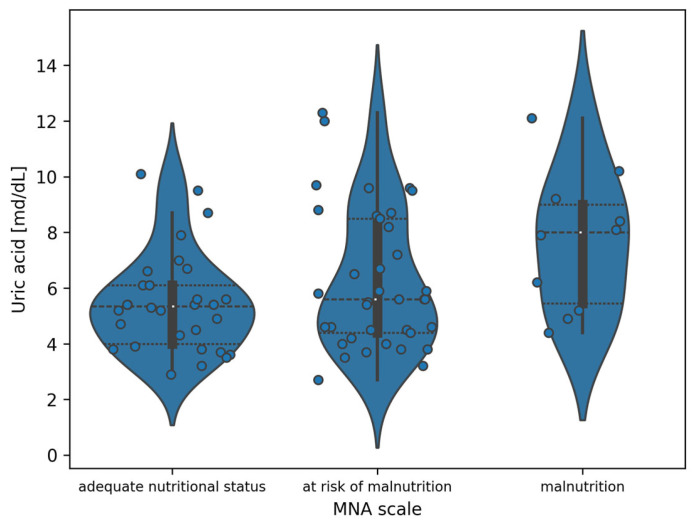
Violin plot of the distribution of uric acid measurement values in the division of patients according to the MNA (white dots indicate the median value).

For the indicated division, analyzing Figure 3, the observed differences are the greatest between the adequate nutritional status and malnutrition groups. To demonstrate the significance of differences between uric acid values in subgroups designated by the MNA, Tukey’s post hoc tests were performed with correction for multiple testing. Full results are included in Table 6. Statistically significant differences were demonstrated between the adequate nutritional status and malnutrition subgroups (*p* = 0.0251)

## 4. Discussion

The influence of uric acid concentration on the daily functioning of older adults is not fully understood. There are conflicting data on its role in basic activities of daily living, dementia nutrition, or depression. Whether uric acid (UA) is a causative factor or a compensatory mechanism in poor health is unclear. The nature of this relationship depends on the dose and the specific disease entity. The question of uric acid’s pro-inflammatory and anti-inflammatory properties remains unresolved, as the results of studies in this area are contradictory. The findings of the present research sit within this broader “pendulum”; moderate uric acid levels have been linked to antioxidant neuro-protection, whereas sustained elevations promote pro-oxidant, pro-inflammatory pathways that may accelerate vascular and cognitive aging; resolving this paradox will require longitudinal designs and mechanistic studies capable of identifying the tipping-point at which UA’s role reverses [4,27].

In the present investigation, ADL scores did not differ significantly between the study groups. However, the study by Wu Y. et al. indicates that higher serum uric acid levels may have a beneficial effect on muscle function due to its antioxidant effect [28]. Moreover, a relationship has been demonstrated between high uric acid levels and poor physical fitness in older persons. An example is the study by B.T. Burke, who analyzed the relationship between gout and hyperuricemia and the results of tests assessing grip strength, the Short Physical Performance Battery (SPPB), and 4 m walking speed. It was found that older people with gout more often had decreased lower limb function. In contrast, upper limb strength remained unchanged. This is consistent with the typical location of joint changes in this disease. In addition, a nonlinear relationship was demonstrated between uric acid levels and walking speed—both low and high levels were associated with an increased risk of slowed gait [29]. A similar relationship was demonstrated between different uric acid levels in the study by C. Ruggiero. The researchers assessed the relationship between UA levels and antioxidant levels and between UA and physical fitness indices, which are generally considered good indicators of global health status in the geriatric literature. An inverse correlation was found between oxidants and UA levels. Their conclusions suggest that UA behaves more like a pro-inflammatory than an antioxidant compound. Furthermore, a detrimental effect of UA on physical fitness and IADL disability was indicated, with a nonlinear distribution—participants with UA levels between 4.8 and 5.3 mg/dL tended to have less IADL disability and higher physical fitness than those with higher or lower UA levels [30]. The study by A. Laudisio et al. noted that elevated uric acid levels are a risk factor for incident osteoarthritis, affecting reduced physical fitness in older patients [31]. The results of their study indicate that serum uric acid (SUA) levels are associated with disability in instrumental activities of daily living in older people. In addition, SUA levels above 5.5 mg/dL can be used in clinical practice to identify older adults who should receive exercise and diet to help prevent the development of disability. Similarly, the study by Min-Gu Kang et al. indicated that elevated circulating UA levels may act as a pro-aging rather than anti-aging factor in older adults, highlighting its potential role in accelerating biological aging [32]. The data further support the utility of serum UA as a potential biomarker of frailty in this demographic group, contributing to the growing body of evidence for its importance in geriatric health assessment. The study by M. Winder et al. identified several factors that contribute to increased mortality in individuals with both low (4–5 mg/dL), very low (<4 mg/dL), and elevated (6–8 mg/dL) or very high (>8 mg/dL) SUA levels [33]. The highest prevalence of malnutrition (MNA ≤ 7 points) and dependence on activities of daily living (ADL ≤ 4 points) were characteristic of the group with the lowest SUA concentration. Similarly, a low ADL score was also observed in the group with the highest SUA concentration.

The present study initially observed a difference in the MMSE score between the normal range and moderate dementia subgroups. However, further statistical tests did not allow for statistical significance. Some papers in the literature present heterogeneous results, such as the study by Asfar et al., which showed increased cognitive dysfunction with increasing serum UA (*p* = 0.019), but post hoc analysis did not show a linear trend [34]. Only the subjects with a uric acid concentration in the lowest and highest quarters differed in Standardized Mini-Mental State Examination (SMMSE) results (*p* = 0.025). Univariate analysis showed a weak but statistically significant relationship between the SMMSE result and UA concentration, whereas in a study of 1598 individuals with a mean age of 72.4 years and UA level of 273.7 ± 70.4 µmol/L, an increased risk of dementia was demonstrated at a level of ≥345 µmol/L for men and ≥292 µmol/L for women after adjustment for cardiovascular risk factors and drugs affecting SUA, NSAIDs or inflammatory markers [35]. A stronger association was demonstrated between vascular or mixed dementia and SUA level (*p* > 0.022) compared to AD-related dementia (*p* = 0.06). Still, this result is unreliable due to the small number of vascular dementias. In contrast, an older prospective cohort study assessing the association between UA level and cognitive function after an average of 11 years showed a reduced risk of dementia and better cognitive function with higher UA levels [2]. The analysis was adjusted for age, sex, and cardiovascular risk factors. Another study found an association between high baseline serum UA levels and rapid cognitive decline [36]. Still, it was not associated with a higher risk of dementia after adjustment for cardiovascular risk factors. Additionally, discrepancies and limitations in the study results may be due to using a single SUA measurement, which makes it impossible to exclude potential time-varying effects of UA.

In recent years, there has also been growing interest in the association of UA levels with depression. Some studies suggest that, regardless of gender, higher UA levels were associated with depressive symptoms [37]. Others, however, have presented opposite conclusions [38]. Moreover, another study showed that men in the highest quartile of UA had a reduced incidence of depression compared with the lowest quartile. This relationship was not seen in women. In addition, there was no correlation between hyperuricemia and a higher incidence of depression compared with its usual level [39]. The present study found no significant association between depression and serum UA levels.

Meta-analytic evidence increasingly supports a dementia-subtype split; pooled prospective data show that each 1 mg/dL rise in serum uric acid is associated with ~12% lower odds of Alzheimer’s disease but ~15% higher odds of vascular dementia [3,40]. Mechanistically, UA can scavenge peroxynitrite and shield amyloid-β-challenged neurons, yet it may also precipitate endothelial dysfunction, oxidative burst, and microvascular inflammation that accelerate cerebrovascular injury. In Poland—where cardiovascular mortality, though declining, remains among the highest in the EU—this provascular pathway could plausibly dominate [41]. The ACE-III–based classification cannot differentiate AD from VaD with sufficient fidelity; neuroimaging, cerebrospinal biomarkers, and small-vessel disease indices would be required. We therefore advocate longitudinal Polish cohorts that pair repeated UA measures with MRI-based subtype adjudication (e.g., white-matter-hyperintensity burden, lacunes) and stratify analyses by evolving cardiovascular-risk trajectories. Such work could clarify whether tailoring UA-lowering therapy is advisable for individuals at high vascular dementia risk while preserving the potential antioxidant benefit for those on an Alzheimer’s trajectory.

This single-center, cross-sectional study relied on a single-serum UA measurement and a modest sample (n = 77). The small sample size limited statistical power—especially for post hoc pairwise MMSE contrasts—and prevented multivariable adjustment. So, non-significant ADL, GDS, and MMSE findings (<0.40 power) may represent type-II error. Consequently, the associations we report should be viewed as correlative signals rather than evidence of causality. Several unmeasured or partially measured confounders could bias the results: detailed renal indices, polypharmacy burden, dietary purines, and low-grade inflammatory markers (e.g., hs-CRP, IL-6) were not collected; successful urate-lowering therapy may coincide with broader comorbidity management, introducing treatment-selection bias. Scale-specific constraints include possible misclassification from the Polish ACE-III cut-off (≤88, whereas 76–82 is used elsewhere), limited nutritional granularity with the three-level MNA, and insufficient numbers for reliable sex-stratified ADL analyses. Although all participants were community-dwelling and electively referred, short hospital stays can induce transient deconditioning; assessments performed in the final ward days may therefore under- or overestimate baseline function, limiting generalizability to purely outpatient older adults. To clarify temporal and mechanistic relationships, future multicenter studies should enroll at least 150–200 older adults, incorporate repeated UA and biomarker panels over 6–12 months, and apply subtype-specific dementia diagnostics. Such work will be essential to determine whether serum uric acid can serve as a practical indicator of depression, dementia, or functional decline in aging populations.

## 5. Conclusions

In summary, although the mechanisms related to uric acid’s influence on older adults’ functioning require further research, the available evidence indicates the relationship between elevated uric acid levels and cognitive decline, as well as deterioration of the nutritional status of older individuals. In clinical practice, uric acid should be regularly monitored in older patients. Moreover, it may be considered a potential indicator of functional decline associated with aging. Nevertheless, further studies are also needed to clarify the association of these uric acid levels with cognitive functions, dementia, and depression.

## Figures and Tables

**Table 1:** Descriptive statistics of quantitative variables in the division of patients into groups.

		Patients Properly Treated for Hyperuricemia	Control Group	Patients with Elevated Uric Acid Levels
Age	N	25	29	23
	mean	81.40	81.93	83.83
	std	8.46	8.46	9.79
	min	63	68	61
	Q1	77	75	79
	median	85	83	87
	Q3	87	90	90
	max	94	94	98
Uric acid	N	25	29	23
	mean	4.73	4.89	9.21
	std	1.08	1.00	1.43
	min	2.7	2.9	7
	Q1	3.8	4	8.3
	median	4.7	4.9	8.8
	Q3	5.6	5.6	9.65
	max	6.7	6.7	12.3
ADL	N	24	29	23
	mean	4.29	4.28	4.17
	std	1.20	1.07	1.30
	min	2	2	1
	Q1	3	4	3.5
	median	5	4	5
	Q3	5	5	5
	max	6	6	6
MMSE	N	25	29	23
	mean	23.40	24.86	22.04
	std	4.37	3.64	4.22
	min	14	16	14
	Q1	21	23	20
	median	24	27	24
	Q3	27	28	24.5
	max	29	29	28
ACE-III	N	25	29	23
	mean	76.92	81.48	71.65
	std	11.94	9.36	9.73
	min	52	62	54
	Q1	74	74	65.5
	median	78	84	72
	Q3	86	88	77
	max	94	92	88
MNA	N	25	29	23
	mean	21.76	22.62	19.78
	std	3.59	4.22	4.10
	min	15	15	15
	Q1	19	19	16.5
	median	22	24	19
	Q3	25	26	22
	max	28	28	29
GDS	N	25	29	23
	mean	9.96	9.21	10.30
	std	4.09	4.30	3.77
	min	4	2	3
	Q1	6	6	7.5
	median	10	10	10
	Q3	12	12	13
	max	19	19	17

**Table 2:** Table of frequencies of geriatric scale classes among patients divided into groups (statistically significant results are highlighted in green).

		Overall	Patients Properly Treated for Hyperuricemia	Patients with Elevated Uric Acid Levels	Control Group	*p*-Value *	Power
N		77	25	23	29		
ADL, n (%)	None	1 (1.3%)	1 (4.0%)			*0.955*	*0.3641*
	independent	39 (50.6%)	13 (52.0%)	12 (52.2%)	14 (48.3%)		
	moderate impairment	29 (37.7%)	9 (36.0%)	8 (34.8%)	12 (41.4%)		
	very dependent	8 (10.4%)	2 (8.0%)	3 (13.0%)	3 (10.3%)		
MMSE, n (%)	cognitive impairments without dementia	18 (23.4%)	6 (24.0%)	8 (34.8%)	4 (13.8%)	*0.160*	*0.6575*
	mild dementia	20 (26.0%)	6 (24.0%)	6 (26.1%)	8 (27.6%)		
	moderate dementia	12 (15.6%)	5 (20.0%)	5 (21.7%)	2 (6.9%)		
	normal range	27 (35.1%)	8 (32.0%)	4 (17.4%)	15 (51.7%)		
ACE-III, n (%)	likely dementia	42 (54.5%)	14 (56.0%)	18 (78.3%)	10 (34.5%)	0.007	0.9033
	normal range	35 (45.5%)	11 (44.0%)	5 (21.7%)	19 (65.5%)		
MNA, n (%)	adequate nutritional status	30 (39.0%)	9 (36.0%)	5 (21.7%)	16 (55.2%)	*0.044*	*0.3561*
	at risk of malnutrition	37 (48.1%)	15 (60.0%)	12 (52.2%)	10 (34.5%)		
	malnutrition	10 (13.0%)	1 (4.0%)	6 (26.1%)	3 (10.3%)		
GDS, n (%)	mild depressives	40 (51.9%)	13 (52.0%)	12 (52.2%)	15 (51.7%)	0.999	0.0501
	normal	37 (48.1%)	12 (48.0%)	11 (47.8%)	14 (48.3%)		

* *p*-values in standard font indicate results from chi-square tests of independence; *p*-values in italics indicate results from Fisher–Freeman–Halton exact tests (FFH).

**Table 3:** Results of post hoc tests (statistically significant results are highlighted in green).

Group A	Group B	Geriatric Scale	*p*-Value	Power
control group	patients with elevated uric acid levels	ACE-III	0.0043	0.8902
control group	patients properly treated for hyperuricemia	ACE-III	0.1833	0.2651
patients with elevated uric acid levels	patients properly treated for hyperuricemia	ACE-III	0.1833	0.2800
control group	patients with elevated uric acid levels	MNA	0.0302	0.7065
control group	patients properly treated for hyperuricemia	MNA	0.1304	0.4222
patients with elevated uric acid levels	patients properly treated for hyperuricemia	MNA	0.0301	0.7806

**Table 4:** Results of ANOVA and Kruskal–Wallis tests. Areas highlighted in gray contain results not included in the analysis due to failure to meet assumptions (ANOVA) or inclusion of a more decisive test (Kruskal–Wallis) (statistically significant results are highlighted in green).

Geriatric Scale	ANOVA				Kruskal–Wallis		
	F	*p*-Value	np2	Power	H	*p*-Value	Power
ADL	0.3726	0.6903	0.6334	0.4209	0.6334	0.7286	0.3947
MMSE	2.5450	0.0626	8.0365	0.9996	8.0365	0.0453	0.9947
ACE-III	15.4842	0.0002	15.9341	1.0000	15.9341	0.0001	0.9999
MNA	3.6647	0.0304	5.4843	0.9997	5.4843	0.0644	0.9933
GDS	0.0572	0.8117	0.0250	0.0874	0.0250	0.8744	0.0845

**Table 5:** Results of post hoc tests of uric acid values divided into groups defined by the MMSE.

Group		U	*p*-Value	Cohen Effect Size
A	B			
cognitive impairments without dementia	mild dementia	171	0.7923	−0.0168
cognitive impairments without dementia	moderate dementia	85.5	0.5272	−0.3154
cognitive impairments without dementia	normal range	305	0.3146	0.5311
mild dementia	moderate dementia	102	0.5946	−0.3328
mild dementia	normal range	371	0.0913	0.6006
moderate dementia	normal range	246	0.0660	0.9480

**Table 6:** Results of post hoc tests of uric acid values divided into groups defined by the MNA (statistically significant results are highlighted in green).

A	B	Mean(A)	Mean(B)	Diff	Se	T	*p*-Tukey	Cohen Effect Size
adequate nutritional status	at risk of malnutrition	5.47	6.25	−0.78	0.55	−1.42	0.3367	−0.3540
adequate nutritional status	malnutrition	5.47	7.66	−2.19	0.82	−2.67	0.0251	−1.1059
at risk of malnutrition	malnutrition	6.25	7.66	−1.41	0.80	−1.76	0.1918	−0.5633

## Data Availability

Data are available on request due to privacy/ethical restrictions.

## Abbreviations

The following abbreviations are used in this manuscript:

ACE-III	Addenbrooke’s Cognitive Examination III
ADL	Activities of Daily Living
FFH	Fisher–Freeman–Halton exact test
GDS	Geriatric Depression Scale
IADL	Instrumental Activities of Daily Living
MMSE	Mini-Mental State Examination
MNA	Mini Nutritional Assessment
NSAIDs	Nonsteroidal anti-inflammatory drugs
SMMSE	Standardized Mini-Mental State Examination
SPPB	Short Physical Performance Battery
SUA	Serum Uric Acid
UA	Uric Acid
